# Increased survival of honeybees in the laboratory after simultaneous exposure to low doses of pesticides and bacteria

**DOI:** 10.1371/journal.pone.0191256

**Published:** 2018-01-31

**Authors:** Franziska Dickel, Daniel Münch, Gro Vang Amdam, Johanna Mappes, Dalial Freitak

**Affiliations:** 1 Centre of Excellence in Biological Interactions, Department of Biological and Environmental Science, University of Jyvaskyla, Jyvaskyla, Finland; 2 Faculty of Environmental Sciences and Natural Resource Management, Norwegian University of Life Sciences, Aas, Norway; 3 School of Life Sciences, Arizona State University, Tempe, United States of America; 4 Centre of Excellence in Biological Interactions, Department of Biosciences, University of Helsinki, Helsinki, Finland; University of California San Diego, UNITED STATES

## Abstract

Recent studies of honeybees and bumblebees have examined combinatory effects of different stressors, as insect pollinators are naturally exposed to multiple stressors. At the same time the potential influences of simultaneously occurring agricultural agents on insect pollinator health remain largely unknown. Due to different farming methods, and the drift of applied agents and manure, pollinators are most probably exposed to insecticides but also bacteria from organic fertilizers at the same time. We orally exposed honeybee workers to sub-lethal doses of the insecticide thiacloprid and two strains of the bacterium *Enterococcus faecalis*, which can occur in manure from farming animals. Our results show that under laboratory conditions the bees simultaneously exposed to the a bacterium and the pesticide thiacloprid thiacloprid had significant higher survival rates 11 days post exposure than the controls, which surprisingly showed the lowest survival. Bees that were exposed to diet containing thiacloprid showed decreased food intake. General antibacterial activity is increased by the insecticide and the bacteria, resulting in a higher immune response observed in treated individuals compared to control individuals. We thus propose that caloric restriction through behavioural and physiological adaptations may have mediated an improved survival and stress resistance in our tests. However, the decreased food consumption could in long-term also result in possible negative effects at colony level. Our study does not show an additive negative impact of sub-lethal insecticide and bacteria doses, when tested under laboratory conditions. In contrast, we report seemingly beneficial effects of simultaneous exposure of bees to agricultural agents, which might demonstrate a surprising biological capacity for coping with stressors, possibly through hormetic regulation.

## 1. Introduction

Agricultural crops are essential for the production of human food as well as being important components for animal feed [1]. It is estimated that approximately 75% of the globally produced food crops for human nutrition rely on pollinating animals [1–3], of which honeybees are economically most important [1]. Agricultural intensification has increased the exposure of pollinators to pesticides [4,5]. Worldwide, around 2.8 million tons of pesticides are applied in conventional farming per year, and approximately 30% of these are insecticides [6,7]. At the same time fertilizers based on microbe-rich manure of farm animals are also widely used [8,9]. Pollinators most likely encounter both pesticides and microbes during their foraging flights when approaching the plants for nectar and pollen collection. Hence it is important to understand how honeybees can cope with multiple stressors simultaneously, even when they encounter them in low doses.

Insecticides are one of the major risk factors for honeybees and other insect pollinators [10], and together with other stressors they might be possible drivers of pollinator population declines in the last decades [5,11,12]. Adverse effects are typically reported as lethal doses in acute exposure tests (e.g. 1-day-LD50), but also as sub-lethal effects after acute exposure and further as chronic exposure (e.g. ≥10 day exposure; [13–15]). The effect of high insecticide doses is well studied and established as toxic to both pest and beneficial insects. However, low doses are common to occur in field crops and other plants, as the insecticide degrades after initial application [16]. This exposure to low, sub-lethal doses can affect behavioural integrity including learning function [17–21], orientation and foraging [19,22–27]. The latter one might be affected by an altered thermoregulation ability of honyebees caused by neonicotinoids or other insecticides [28,29]. Apart from behavioral effects, sub-lethal doses of neonicotinoids can also affect the insects physiology [30]. Increased susceptibility to microbiological pathogens like *Nosema* has also been documented after sub-lethal pesticide exposure [31–33].

Globally, neonicotinoids are the most extensively used insecticide class, which account for up to one third of the insecticide market [34]. They are systemic, whereby a direct uptake by growing crops enables more indirect application methods, e.g. by seed coating [34]. This characteristic can eliminate the need for spraying pesticides during pollination season [32], hence they are considered to be less harmful for beneficial insects [34]. However, due to their high solubility and persistence these pesticides can stay available in the soil, are taken up by plants over long periods of time [35], and consequently can end up in pollen and nectar, posing risks for foraging pollinators [32,36–38].

Concerns about the intensification of agriculture and the effect of pesticides on human and animal health have led to more precise guidelines and restrictions for the application of pesticides during the last years [39]. In addition more sustainable farming methods have raised attention [22–24,40–42]. Conventional farming differs from the more sustainable organic farming methods for example in their application of synthetically produced insecticides and artificial fertilizers [42,43]. In organic farming synthetically produced pesticides are not approved and the use of natural fertilizers such as compost and manure, which are rich in various bacterial species [44], are a common practice. These includes the gram positive, opportunistic pathogen *Enterococcus faecalis* [44,45], a very persistent pathogen that can be found in plants [46]. In lepidoptera, *E*. *faecalis* strains can be virulent [46,47], whereas in the same host *E*. *faecium* only shows very low virulence [46]. It is documented that *E*. *faecalis* and *E*. *faecium* are harboured by beetles and flies [48] and Martin and Mundt (1972) showed that Enterococci species occur more often in nectar-feeding insects than in leaf or stem piercing insects [49]. When manure gradually decomposes, nutrients and also bacteria are released into soil and water. Plant roots then take them up, transporting them to other plant tissues via xylem and phloem transport [50]. Potential bacterial pathogens may thus also afflict pollinators, which collect pollen and nectar from affected field sites during their foraging. Indeed, *E*. *faecium* strains have been found in the guts of *Apis mellifera* adults [51], which confirms the ability of *Enteroccous* strains to persist in the intestinal tract of honeybees. However, it is still largely unknown whether biological fertilizers might actually have a possible adverse effect on insect health.

The foraging areas of bees are naturally rather big [52,53]. It is also known that there can always be a drift of insecticides during application or through pollen flight from already contaminated plants. The application of neonicotinoids is not allowed in organic farming, but they can still contaminate organic farmed field sides due to drift during application or pollen flight [54,55]. In addition, neonicotinoids are very persistent in soil and accumulate in ground water, which is why they could still contaminate plants years after their actual application [56–59]. Manure and organic fertilizers, on the other hand, are widely used in conventional and organic farming. These facts suggest that bees may encounter various agricultural stressors simultaneously during their foraging flights. Recent studies have revealed that combinations of different stressors can cause more harm to organisms than single stressor exposure [60]. For example, the synergistic effects of parasites, pesticides and predation result in increased mortality in the ecotoxicology model *Daphnia magna* [61], insecticides show additive negative effects on pollinator health by reducing the number of workers, and the foraging efficiency in bumble bees [62], and can impair foraging behaviour in honeybees [20]. Furthermore, exposure to a pesticide can increase bees susceptibility to parasites, like the gut pathogen Nosema [31,33,63,64], and combined exposure to neonicotinoids and parasites reduce bumble bee queen survival [65]. The effect of simultaneously occurring chemical and biological (bacterial) stressors on pollinators still remains largely unknown.

Insects have evolved a great variety of defence mechanisms to protect themselves against diseases and parasites [66,67]. The main physiological defence barrier, the innate immune system [68,69], consist of well-evolved immune responses, the humoral and cellular immunity. Insect haemolymph contains different types of haemocytes which have several major functions in immune responses, like phagocytosis, encapsulation, nodule formation and the production of reactive oxygen species, e.g. through the activation of phenoloxidase [68,70–74]. Social insects like honeybees have also evolved a collective defence system against parasites, the so called social immunity, which is based on cooperation between the colony members to avoid, eliminate or control infections [75].

Here we studied the effect of three agricultural stressors on honeybee health using a carefully controlled and full factorial experimental design conducted under laboratory conditions. The aim was to test whether sub-lethal doses of single biological and chemical agricultural stressors have an additive or synergistic negative effect on individual pollinator survival and immunity when encountered simultaneously. Honeybees were orally exposed to the insecticide thiacloprid, representing a chemical stressor, two strains of the bacterium *Enterococcus faecalis*, which are found in animal faeces and organic fertilizers, as well as a mixture with all of them. To assess potentially adverse effects, we monitored survival combined with two standard tests for immunocompetence and feeding quantity.

## 2. Material and methods

### 2.1 Animals

The experiments were performed at the Norwegian University of Life Sciences (Aas, Norway) with the honeybee *Apis mellifera carnica*. Sealed brood with late pupa stages were collected from three different donor colonies and kept for approximately two days in an incubator at 34°C. In total ca. 2.400 freshly emerged adults were then marked on the thorax with a paint spot. The bees were then re-introduced into two host colonies, different from the donor colonies they were collected from, to ensure similar exposure to a non-native environment. This was done to ensure that newly hatched bees, which are mostly devoid of gut bacteria, receive microbiota through initial feeding by older siblings (trophallaxis) [76]. After seven days, matured bees were re-collected from the host colonies, and placed in plastic cages lined with mesh (cage dimensions with width/depth/height: 19/18/23 cm). Cages with adult bees were kept in an incubator at a lower temperature of 26°C (representing the temperature of other colony environments than the brood chambers) with approximately 60% relative humidity [77]. They were offered water and sucrose (50% v/v bifor^TM^ (http://www.nordicsugar.com/animal-feed/bees/biforr/), 1% lipid mix (L5146, Sigma), 2% amino acid mix (R7131, Sigma), 22% water) *ad libitum* via plastic syringes (10 mL) attached to the top of the plastic cage.

### 2.2 Bacteria and insecticide

*Enterococcus* bacteria are a group of gram-positive opportunistic pathogens, naturally found in the gastrointestinal tracts of mammals and insects [45], but at the same time can also cause intestinal and urinal infections [78,79]. Being very resilient they can stay viable in different environments, and thus can be found in soil, sand, water, as well as on plants [46,79]. *Enterococcus faecalis* is one of the best studied bacterial species and is naturally abundant through contamination by faeces [79], and is documented to occur in 37 different insect taxa [45]. This bacterium predominantly inhabits animal-associated environments but also is an important infectant in humans, possessing virulence factors such as gelatinase [46] and can be spread by contamination with faeces [79]. Two strains of *Enterococcus faecalis* were used for this experiment: MMH594 (MMH) which is highly virulent strain (measured as survival of *Ceanorhabditis elegans* feeding on *E*. *faecalis*) isolated from human blood, and FLY1 (FLY) which was isolated from *Drosophila* [80]. The bacteria were cultivated in GM-17 growing medium (37.25 g M-17 Broth (#56156, Sigma), 50 mL sterilized 10% glucose in 950 ml dH_2_O) and M-17 Agar (48.25 g M-17 Agar (#63016, Sigma) in 950 ml dH_2_O). The culture was then washed with PBS (phosphate buffered saline) and the cell pellet diluted in sucrose to an optical density of 0.5 at 600nm (approximately 2.5 x 10^8^ cells/ml), which was used as bacteria treatment for the experiment. The effects of both strains on honeybee survival (see S1A Fig) and immune response (measured as lytic activity) was tested with two concentrations (see S2 Fig). Thiacloprid was obtained as a dry powder (100%, #37905, Sigma), prepared in DMSO and diluted in sugar syrup to obtain a final concentration of 50% sucrose, 0.1%DMSO, 2% amino acids, 1% lipids and 3.78μg/ml thiacloprid. The final concentration was determined so that bees approximately ingested a daily dose of 1/100^th^ of LD50 (LD50 in bees = 17 μg/bee according to Vidau et al. [41]). We exposed bees to an approximate individual dose of 0.17μg/0.044ml (45 bees ingest approximately 2ml food per day, 1 bee ingests 0.044ml food per day). This dose resulted in a sub-lethal effect of survival in our pre-experiments (see S1B Fig). Sub-lethal concentrations are usually considered as safe for non-target organisms in agricultural pest control [81]. However, thiacloprid residues can vary greatly depending on the plant species or the plant part, but also within the same sampling part. *Pohorecka et al*. *for example showed* much smaller insecticide concentration in nectar sampled from flowers (mean concentration *6*.*5μg/g*, *max*. 208.8μg/g) than in pollen samples from pollen traps (*mean concentration 89*.*1μg/g*, max. 1002.2μg/g) of oilseed rape [82]. Samples were taken during florescence, which also represents the time when foraging bees would be exposed to the insecticide residues. The from GAP (Good Agricultural Practice) authorized application rate for thiacloprid is 72g/ha for rapeseed [83]. Furthermore, pesticides need to undergo strict tests during their registration process and are only permitted for commercial use when fulfilling strict safety guidelines for human and environment (non-target organisms) protection [84]. We thus concluded that a sub-lethal dose of 0.17μg/bee represents a field realistic dose, even ranging under the in nectar occurring doses. The toxicity of the LD50 dose of thiacloprid was established in pre-experiments by oral feeding of adult bees (see S1B Fig).

### 2.3 Experimental set-up

Re-collected bees from the host hives were kept for 24 h in an incubator with standard food and water ad libitum. A full factorial experiment was conducted, using thiacloprid as the insecticidal stressor, the *E*. *faecalis* strains MMH and FLY as bacterial stressors, sugar solution as control treatment, and the four possible treatment combinations (thiacloprid+MMH, thiacloprid+FLY, MMH+FLY, thiacloprid+MMH+FLY) as multiple stressors, summing up to eight treatment groups. The bees were grouped into 45 individuals per cage, including individuals from each colony, and four replicates for each treatment. In total 32 cages and 1440 bees were used for the experiment. Every day, for a total of eleven days the cages received freshly prepared dietary treatments (6 mL, thiacloprid = 3.78μg thiacloprid per ml food, bacteria = 2ml food with 0.5 OD (approximately 2.5 x 10^8^ cells/ml)) for details see Section 2.2 above) to obtain a chronically constant exposure during the experimental period. The diet was offered via plastic syringes inserted in the top of each cage, in addition each cage was offered water ad libitum via a second syringe.

### 2.4 Monitoring of survival and feeding

Long-term cage experiments may cause exponential mortality rates that typically onset after 10–15 days (personal observation), due to stress caused by the artificial beekeeping conditions [15,85]. Studies of about 10 days, on the other hand, might have lower risk of a mortality bias due to stress by the artificial environment. Both methods are constantly improved and are considered as standard for testing chronic toxicity [15,86]. For our study we chose a short-term monitoring period to test an essential healthy cohort and minimize possible stress effect on bees by being captive. In our study bees were exposed to the treatments for a period of 11 days and mortality was monitored daily as well as dead bees counted and removed. Additionally the amount of food intake was monitored every day during the whole experimental period. Feeding was measured in mL of food uptake in 24 h per cage.

### 2.5 Haemolymph sampling

All bee samples were taken 11 days after the first initial inoculation with the treatments. To assess the general bacterial activity 4 μl of fresh haemolymph was collected with a micro capillary (P1424, Sigma) from 16 individuals per treatment group, 4 samples from each cage, totaling 128 samples. For the hydrogen peroxidase assay, the guts of three individuals per cage and treatment were dissected, totaling 96 samples in total. The midgut was preserved at -80°C in 200 μl of 2 mg/ml Amino-triazole (A8056, Sigma) until further usage.

### 2.6 Immunological assays

General antibacterial activity was assessed straight from freshly sampled haemolymph. 4 μl of haemolymph was directly pipetted into 2 mm diameter wells punctured on PBS (phosphate buffered saline) 1% agar plates containing lyophilizised *Micrococcus lysodeikticus* cells (*Micrococcus lysodeikticus* ATCC #4698, Sigma), incubated over night at 37°C and then photographed for later analysis. Lysozyme-like activity was calculated by measuring the radius of the clear zone around each sample. A standard curve was generated by diluting chicken egg white lysozyme (L6876, Sigma) to 7 serial dilutions (2 mg/ml, 1 mg/ml, 0.750 mg/ml, 0.500 mg/ml, 0.250 mg/ml, 0.125 mg/ml, 0.62 mg/ml, and 0.31 mg/ml).

The concentration of reactive oxygen species (ROS) in the gut was measured as an amount of H_2_O_2_ by using the Amplex^®^ Red Hydrogen Peroxide/Peroxidase Assay Kit (life technologies, Eugene, Oregon, US), following the manufacturer’s protocol using a multiplate reader (EnSpire, Perkin Elmer, Waltham, MA, US). Samples were thawed on ice, homogenized, centrifuged and the supernatant transferred for further usage. In order to correct for the possible differences in the amount of gut tissue obtained, the protein concentration of each sample was assessed (Pierce™ BCA Protein Assay Kit, # 23227, Thermo-Fisher Scientific, Waltham, MA, US) and used to calculate the H_2_O_2_ concentration per unit of protein. Two microliters of the sample were used for the assay with two technical replicates for each sample.

### 2.7 Statistics

Statistical analyses were done using SPSS statistics v. 22.0. All analyses were performed with 10 comparisons, focusing on the effect between the different treatments (diets) on mortality, food consumption and immunocompetence. The comparisons of interest are: control group against all treatments (7 comparisons) and the treatment with all three stressors against each single stressor (3 comparisons). All comparisons were conducted using Bonferroni adjusted alpha levels of 0.005 (0.05/10) per test. Mortality was analyzed using Kaplan-Meier survival analysis with a log-Rank test for overall comparison and single log-Rank test for the 10 single comparisons. Differences in the quantity of diet ingested by the honeybees during the experiment were analyzed with ANOVA with treatment as factor and food per capita (amount of food consumed by each bee) as independent variable, comparing the 10 contrasts. The amount of food ingested was calculated per bee per day to correct the mean amount of food ingested for the number of bees in each treatment on each day. General antibacterial activities in haemolymph were analyzed using a Kruskal-Wallis test because of non-normal distribution on the data. Man-Whitney U-test was used as non-parametric post hoc test on the 10 specified comparisons. Differences in ROS concentration in the gut were assessed with ANOVA and following post hoc test on the 10 comparisons.

## 3. Results

### 3.1 Survival

**Fig 1:**
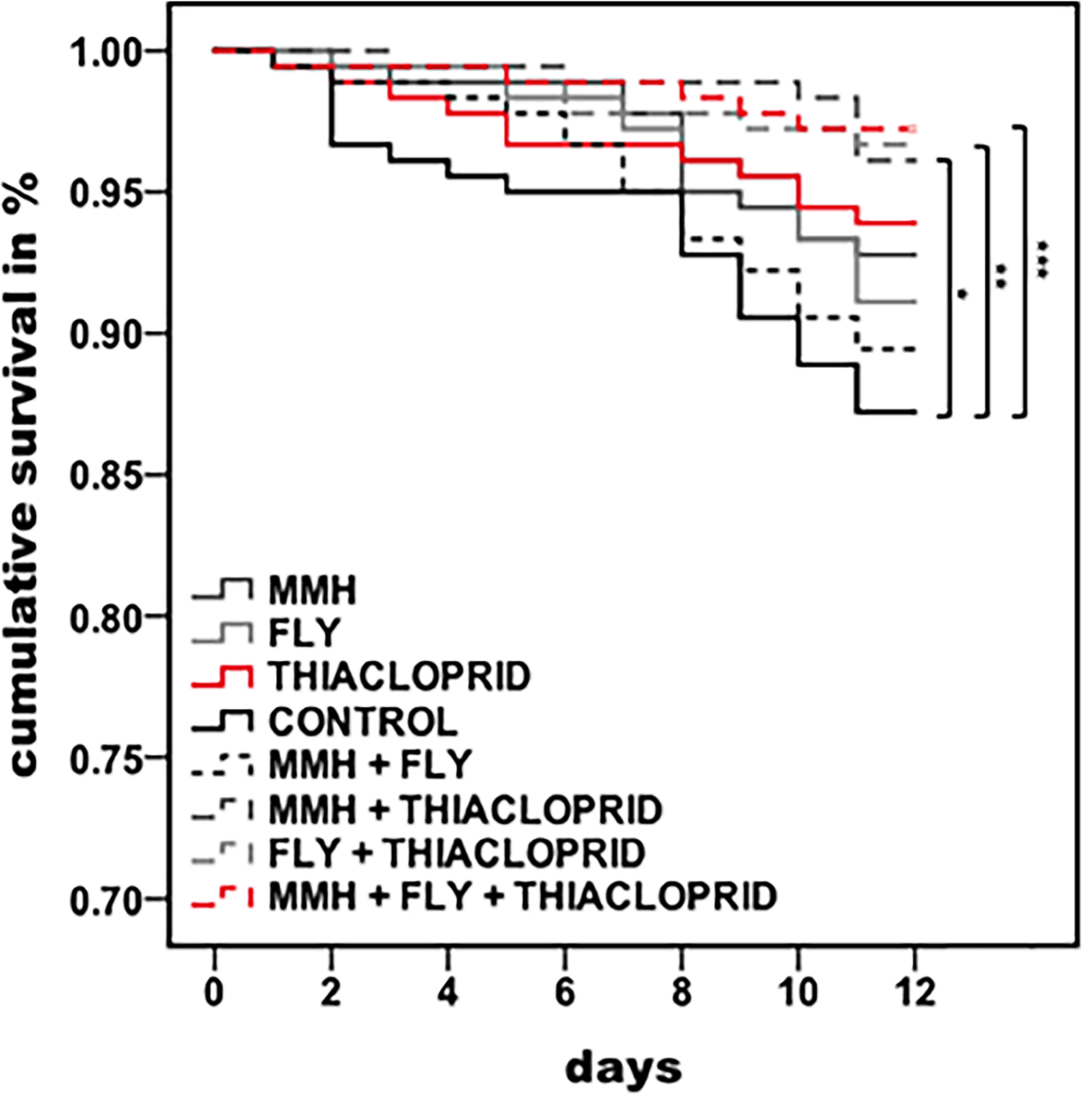
Effect of exposure to single and combined agricultural agents on honeybee survival. Survival is expressed as the percentage of cumulated number of surviving bees during the monitoring period of 11 days for each treatment group with n = 180 (MMH = *E*. *faecalis* strain MMH594, FLY = *E*. *faecalis* strain FLY1). Asterisks indicate differences in the survival rate after 11 days of individuals exposed to different stressors, * ≤ 0.005, ** ≤ 0.001, *** ≤ 0.0001 (Kaplan-Meier survival analysis, log Rank test for overall comparison).

Honeybee survival was overall significantly affected by the agents they orally ingested (Fig 1: Kaplan-Meier log Rank: df = 7, X^2^ = 25.686, p = 0.001). Simultaneous exposure to combinations of low doses of thiacloprid and bacteria increased the honeybees survival compared to the control group (Table 1: multiple log Rank tests, Bonferroni adjusted alpha-level = 0.005). However, the mixture of both bacterial strains did not affect the survival of bees compared to the control individuals, and the survival of bees exposed to single stressors did not differ from survival of bees exposed to a mixture of all three stressors. The ingestion of a mixture containing all three components (bacteria and insecticide) resulted in the highest survival rates after 11 days (survival rates: Control = 87%, MMH+FLY = 89%, FLY = 91%, MMH = 93%, THIA = 94%, MMH+THIA = 96%, FLY+THIA = 97%, MMH+FLY+THIA = 97%).

**Table 1:** Pairwise differences of honeybee worker survival rate 11 days post oral exposure to thiacloprid and *E*. *faecalis* and control diet.

	Chi-square	p-value
**C vs. THIA**	4.618	0.032
**C vs. MMH**	3.108	0.078
**C vs. FLY**	1.524	0.217
**C vs. MMH+FLY**	0.434	0.510
**C vs. MMH+THIA**	9.561	0.002
**C vs. FLY+THIA**	10.752	0.001
**C vs. MMH+FLY+THIA**	12.476	0.0004
**MMH+FLY+THIA vs. THIA**	2.355	0.125
**MMH+FLY+THIA vs. MMH**	3.706	0.054
**MMH+FLY+THIA vs. FLY**	6.003	0.014

MMH, *E*. *faecalis* strain MMH594; FLY, *E*. *faecalis* strain FLY1; THIA, thiacloprid; MMH+FLY, a mixture of both bacterial strains; MMH+THIA, thiacloprid mixed with the bacterial strain MMH594; FLY+THIA, thiacloprid mixed with the bacterial strain FLY9; MMH+FLY+THIA, a mixture of all three components; C, control; Kaplan-Meier log Rank tests with Bonferroni corrected alpha-level of 0.005, n = 180 each treatment group.

### 3.2 Feeding

**Fig 2:**
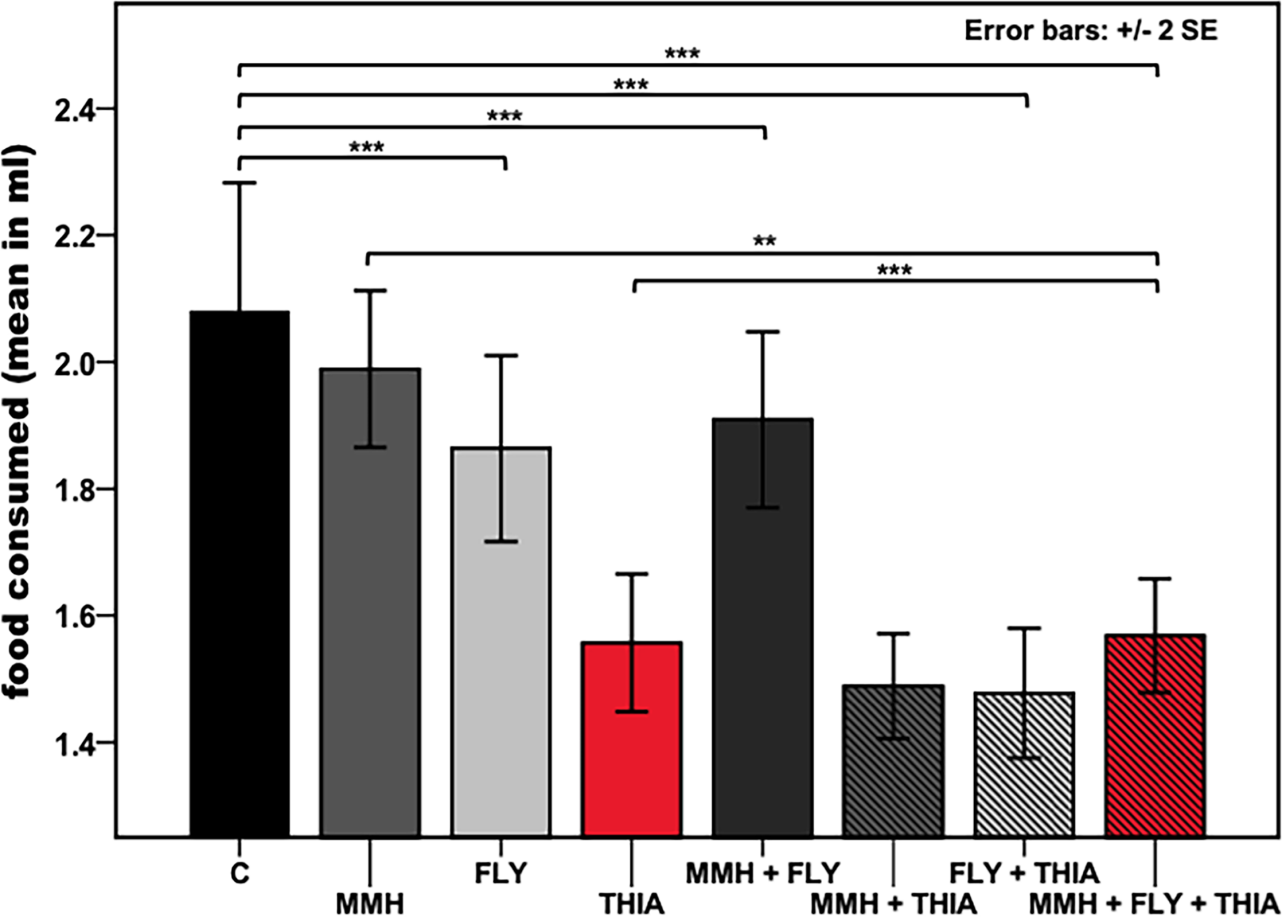
Quantity of food uptake by honeybees depending on the diet. Food consumed representing the average mean amount of diet consumed in ml per bee per day during the 11-day monitoring period. Diets were inoculated with potential agricultural stressors (C = control, MMH = *E*. *faecalis* strain MMH594, FLY = *E*. *faecalis* strain FLY1, THIA = thiacloprid) with n = 1341 bees in total after 11 days (THIA n = 167, MMH n = 164, FLY n = 169, CONTROL n = 157, MMH+THIA n = 161, FLY+THIA n = 173, MMH+FLY n = 175, MMH+FLY+THIA n = 175; food consumption was controlled for bees per diet). Results represent mean values ± 2 s.e.m. Asterisks indicate differences in average food consumed per bee, * ≤ 0.005, ** ≤ 0.001, *** ≤ 0.0001; post hoc comparisons adjusted with Bonferroni (alpha = 0.005). Bars are indicated in: black = control, dark-grey = MMH, light-grey = FLY, red = THIA, shaded black = MMH+FLY, shaded dark-grey = MMH+THIA, shaded light-grey = FLY+THIA, shaded red = MMH+FLY+THIA.

There was a significant difference in the food consumption between different diets (Fig 2: ANOVA: F(7,344) = 17.482, p< 0.001). A diet containing the bacterial strain FLY significantly affected the mean feeding intake of bees compared to the control group (Table 2: ANOVA post hoc test, Bonferroni adjusted alpha-level = 0.005). Feeding on a diet containing all stressors also resulted in significantly decreased feeding intake compared to the control group, and individuals feeding on diet containing only thiacloprid or only the bacterial strain MMH.

**Table 2:** Pairwise differences in feed quantity ingested by honeybee workers between diets inoculated with thiacloprid and *E*. *faecalis*.

	t-value	p-value
**C vs. THIA**	1.345	0.183
**C vs. MMH**	2.326	0.023
**C vs. FLY**	4.953	p< 0.0001
**C vs. MMH+FLY**	6.010	p< 0.0001
**C vs. MMH+THIA**	1.691	0.095
**C vs. FLY+THIA**	6.117	p< 0.0001
**C vs. MMH+FLY+THIA**	5.276	p< 0.0001
**MMH+FLY+THIA vs. THIA**	-5.812	p< 0.0001
**MMH+FLY+THIA vs. MMH**	-3.732	0.0003
**MMH+FLY+THIA vs. FLY**	-0.241	0.810

MMH, *E*. *faecalis* strain MMH594; FLY, *E*. *faecalis* strain FLY1; THIA, thiacloprid; MMH+FLY, a mixture of both bacterial strains; MMH+THIA, thiacloprid mixed with the bacterial strain MMH594; FLY+THIA, thiacloprid mixed with the bacterial strain FLY9; MMH+FLY+THIA, a mixture of all three components; C, control; ANOVA post hoc test with Bonferroni corrected alpha-level of 0.005, n = 1341 bees in total after 11 days (THIA n = 167, MMH n = 164, FLY n = 169, CONTROL n = 157, MMH+THIA n = 161, FLY+THIA n = 173, MMH+FLY n = 175, MMH+FLY+THIA n = 175).

### 3.3 Immunity

**Fig 3:**
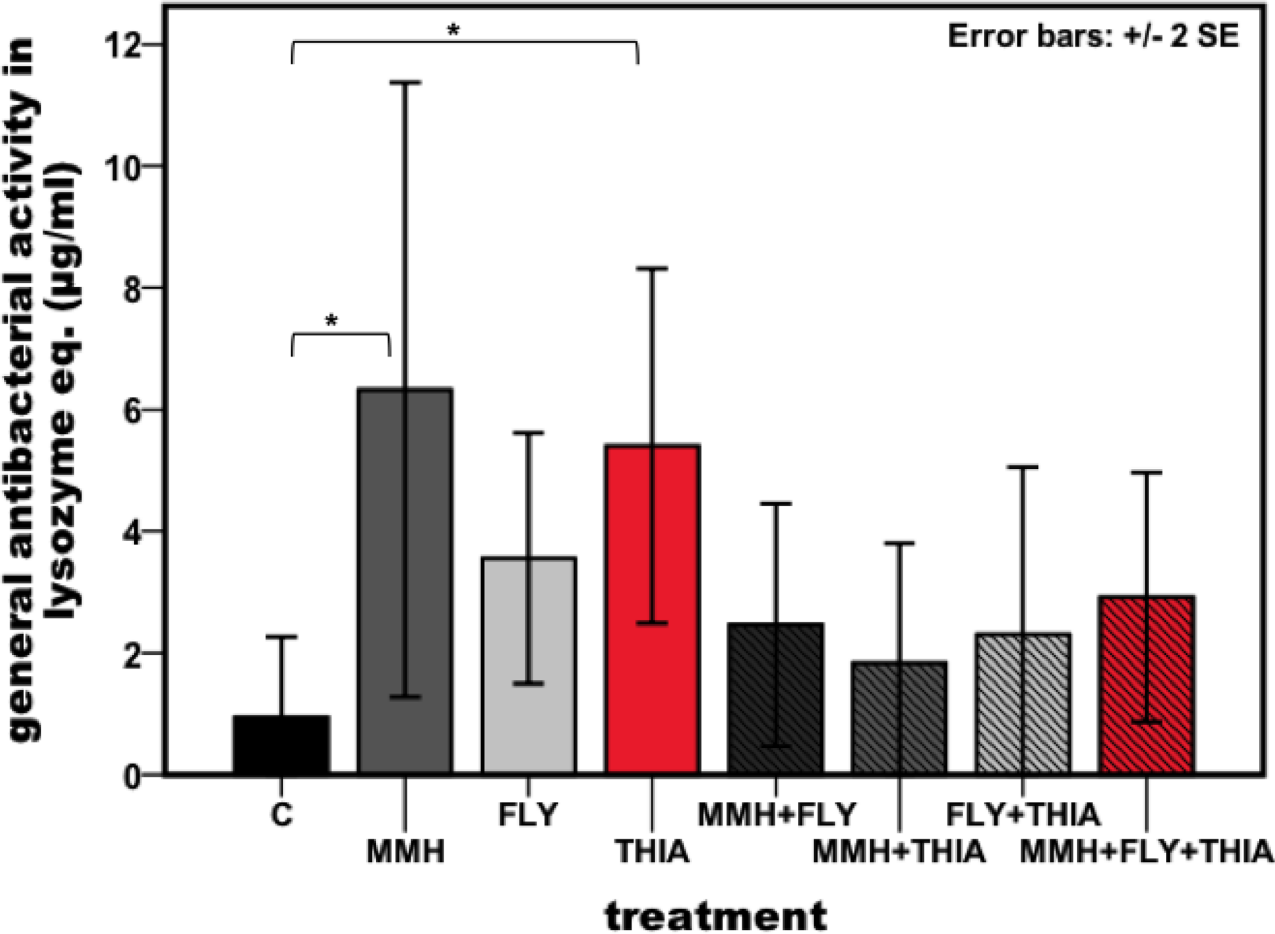
Lysozyme-like activity in the haemolymph of honeybees after being exposed to thiacloprid and *E*. *faecalis*. General antibacterial activity measured as the diameter of the lytic zone on agar plates, transformed to lysozyme equivalents (μg/ml). Different enzyme activity in the haemolymph as a result of all possible different diet combinations as well as single stress exposure (C = control, MMH = *E*. *faecalis* strain MMH594, FLY = *E*. *faecalis* strain FLY1, THIA = thiacloprid). Asterisks indicate differences in the lysozyme-like activity, * ≤ 0.005, ** ≤ 0.001, *** ≤ 0.0001. Results represent mean values ± 2 s.e.m, Kurskal-Wallis test, n = 16 for each treatment group. Bars are indicated in: black = control, dark-grey = MMH, light-grey = FLY, red = THIA, shaded black = MMH+FLY, shaded dark-grey = MMH+THIA, shaded light-grey = FLY+THIA, shaded red = MMH+FLY+THIA.

**Fig 4:**
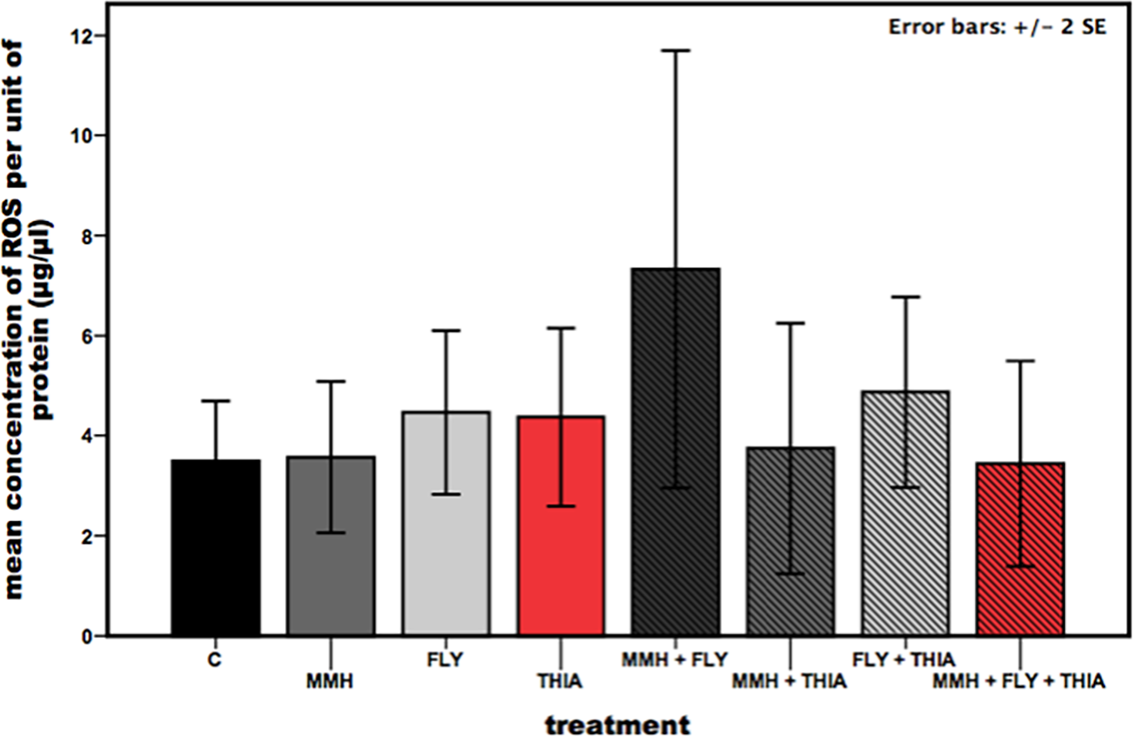
ROS concentration in the gut of honeybees after being exposed to different agricultural agents. Differences in the mean concentration of reactive oxygen species in honeybee midguts, adjusted to the units of protein, when feeding on different diets. Diets were inoculated with potential agricultural stressors (C = control, MMH = *E*. *faecalis* strain MMH594, FLY = *E*. *faecalis* strain FLY1, THIA = thiacloprid). Results represent mean values ± 2 s.e.m, ANOVA, n = 16 for each treatment group. Bars are indicated in: black = control, dark-grey = MMH, light-grey = FLY, red = THIA, shaded black = MMH+FLY, shaded dark-grey = MMH+THIA, shaded light-grey = FLY+THIA, shaded red = MMH+FLY+THIA.

The lytic activity differs significantly between the treatments (Fig 3: Kruskal-Walles test, df = 7, X^2^ = 17.065, p = 0.017). The lysozyme like activity was increased when feeding on the insecticide thiacloprid and also on the single strain MMH of *E*. *faecalis* (mean lytic activity: Control = 0.94±0.662, MMH = 6.32±2.525, FLY = 3.56±1.031, THIA = 5.40±1.457, MMH+FLY = 2.46±0.997, MMH+THIA = 1.83±0.985, FLY+THIA = 2.30±1.377, MMH+FLY+THIA = 2.92±1.025) and was significantly different (Table 3: Mann-Whitney U-test, Bonferroni adjusted alpha-level = 0.005). Ingestion of multiple stressors did not significantly change the lysozyme like activity in the haemolymph compared to control and single treatment exposure. Concentration of ROS in the gut did not differ between all possible treatment combinations (Fig 4: ANOVA: F(7, 83) = 1.239, p = 0.291).

**Table 3:** Pairwise differences in lytic like activity of honeybee workers after feeding on different stressors.

	U	p-value
**C vs. THIA**	62.000	0.004
**C vs. MMH**	60.000	0.004
**C vs. FLY**	69.000	0.011
**C vs. MMH+FLY**	79.000	0.029
**C vs. MMH+THIA**	100.000	0.165
**C vs. FLY+THIA**	112.000	0.376
**C vs. MMH+FLY+THIA**	83.000	0.039
**MMH+FLY+THIA vs. THIA**	78.000	0.044
**MMH+FLY+THIA vs. MMH**	103.000	0.329
**MMH+FLY+THIA vs. FLY**	115.000	0.609

MMH, *E*. *faecalis* strain MMH594; FLY, *E*. *faecalis* strain FLY1; THIA, thiacloprid; MMH+FLY, a mixture of both bacterial strains; MMH+THIA, thiacloprid mixed with the bacterial strain MMH594; FLY+THIA, thiacloprid mixed with the bacterial strain FLY9; MMH+FLY+THIA, a mixture of all three components: C, control; post hoc Man-Whitney U-tests, n = 16 for each treatment.

## 4. Discussion

We found that the oral exposure to a diet containing the sub-lethal dose of two different stressors simultaneously increased honeybee survival compared to control individuals. To our knowledge, there are no other known studies on bees, examining the effect of *E*. *faecalis*. At the same time the influence of multiple stressors, like different pesticides [62,87] or pesticides and pathogens [31,64] have shown negative interactive or synergistic effects on bee health. Our results, that indicate a possible positive effect of simultaneous exposure of two sub-lethal stressors in short term, might provide additional support for studies reporting positive effects of mild stress on life-history traits, like survival, reproduction or immunity, in bees [88] as well as other insects [89,90]. It could be also interpreted as a case of insecticide-induced hormesis, described by Cutler and Guedes [91,92]. This might demonstrate a capability of individual bees to cope with potential stressful agents, but would need further confirmation by subsequent experiments. While *E*. *faecalis* does not kill honeybees at low doses, we did see a slight increase in immune responses against one strain (see supporting information). This might mean, that these effects are specific to a bacterial strain honey bees are encountering and while some strains are benign, others could lead to immunopathology. Similar effects have been documented for enterobacteria in mammalian gut [93]. In addition it is known that *Enterocoocus* strains are able to persist in the gut of bees [51], which is why we assume that it may act as a mild potential stressor to them. Thiacloprid is highly toxic to honeybees in high doses (LD50 = 17 μg/bee according to Vidau et al. [41], see supporting information), and furthermore studies also proved negative effects of sub-lethal insecticide doses in different insect species [13,22,41]. However, the long-term effects of simultaneous exposure to such stressors are not known and so it could still potentially negatively affect behaviour and longevity if the experiments were run for an extended amount of time.

Different dietary treatment did affect the antibacterial activity of honeybees. Compared to control treated individuals the antibacterial activity in the haemolymph was up-regulated in response to a diet with thiacloprid or the bacterial strain MMH. Surprisingly, the exposure to a combination of all stressors did not further increase the immune response. The immune defense is a physiological adaptation evolved to defend the organisms against infections and is shown to be in trade-off with other life-history traits [74,94–96]. In our experiment, the bees might need to invest simultaneously in different physiological responses (e.g. immunity vs. detoxification) to fight against the simultaneous exposure to bacteria and insecticides. Hence, it may be important to keep costs for single processes low in order to maintain the functioning of all system processes [97]. This might be also represented by the high survival rates of individuals exposed to all three stressors simultaneously, which did not show high immune response, indicating a lack of a harmful additive effect by simultaneously occurring sub-lethal stressors. While a down-regulation of immune responses can reduce the costs for individuals, it can impair survival during an ongoing, chronic infection or when encountering additional pathogens. However, in our case the level of antibacterial activity was still higher in honeybees stressed with thiacloprid or with the E. faecalis strain MMH compared to control individuals.

Honeybees feeding on a diet containing thiacloprid did show a significant lower food intake compared to individuals on the control diet and bacteria-only diets. This finding might be in line with results by Kessler et al (2015), which showed that pollinators were attracted to a diet containing neonicotinoids, but the overall food consumption was actually decreased. Caloric restriction and starvation can cause malnutrition, impairment of immune response [98] and thus can negatively affect life-history traits. However, in our study individuals feeding on a diet containing thiacloprid plus bacteria, showing a decreased food intake, did actually show beneficial effects on increased survival rates. Even though the ingestion of only bacteria containing diet did not cause the same results, the response to thiacloprid containing diet might be explained by the evidence that a moderate food- or caloric restriction can have beneficial effects by increasing survival, longevity or resistance against diseases or stress [98]. In addition, the decreased food intake also implies a decreased intake of the insecticide, which might lower its possible negative effect. Still, decreased food consumption also results in a reduced intake of nutrients, which could result in a harmful fitness effect for the bees. A controlled caloric restriction can lead to increased heat-shock protein or antioxidant levels, which both contribute to insects survival by increasing the resistance to stress. Antioxidants protect proteins and DNA from oxidative damage and heat-shock proteins play an important role in insects’ stress response by ensuring protein integrity [99–101]. At this point we can only speculate about how much our results and the beneficial effect of simultaneous exposure to thiacloprid and bacteria could be explained by a caloric restriction driven response. In addition, a decreased food intake by individual worker bees might lead to starvation of the whole colony, as they will bring back less food from their foraging flights.

Furthermore a reduced caloric intake or dietary restriction can influence the dose-dependent response of hormetic agents [98] and can lead to the extended lifespan recorded in rats and mice [102] as well as in insects [103] and increased survival or resistance against diseases [98,99]. Hormetic agents are chemicals or environmental factors, which can result in beneficial effects on biological processes and survival at low doses, while at high doses they are harmful for an organism [88,92,104–106]. Although it is yet to be shown how higher doses introduced for longer periods might affect bees, it is possible that thiacloprid in combination with other stressors might act as a hormetic agent in the short term. This is based on the toxic effect of the LD50 dose of thiacloprid on honeybees (according to S1B Fig) during short-term exposure under laboratory conditions, whereas at low doses it did show a beneficial effect on individual bees under similar conditions. Additional experiments are needed to examine under which conditions the beneficial responses at low doses might switch to adverse outcomes at higher doses–a binary response pattern typical for hormetic responses.

Our aim was to study the effect of the agricultural agents on survival and immune parameters of individuals; thus, we excluded any natural behaviours, like supplementary feeding and flying behaviours, from our experiment. Here we report that even approved and officially considered as “safe” sub-lethal doses of thiacloprid can negatively affect the feeding behaviour of individual pollinators. Although a simultaneous exposure to low doses of stressors did increase survival in our experiment, the hive-level consequences of decreased food intake are yet to be shown. The effect of pesticides on bee health is of increasing importance and is already an established research field. Many studies have investigated the effect of dose dependent toxicity on bee health, and have shown that bees might prefer feeding on insecticide contaminated over uncontaminated diet, while at the same time their overall food intake is reduced [107]. The effect of simultaneously occurring stressors, especially from different origin (chemical and biological) still needs further research. While it would be important to investigate the colony response and their inclusive fitness by testing whether the beneficial effects of exposure to multiple agricultural low dose stressors also manifest under natural conditions, we here show that low doses of simultaneously contacted agricultural stressors seem to confer positive short-term effects of survival on individual level.

## Supporting information

S1 Fig Effect of exposure to different *E*. *faecalis* strains and the insecticide thiacloprid on honeybee survival. Cumulative survival of adult honeybees after (A) oral exposure with two different concentrations (OD 1 and OD 0.5) of the *E*. *faecalis* strains MMH and FLY (Kaplan-Meier log Rank: df = 4, X^2^ = 1.603, p = 0.808), and (B) oral exposure of the LD50 dose of the insecticide thiacloprid as well as two lower doses (1/50^th^ of LD50 and 1/100^th^ of LD50) (Kaplan-Meier log Rank: df = 3, X^2^ = 979.480, p = 0.000; multiple comparison at day 2 for LD50: LD50 vs. 1/50^th^ of LD50 p<0.000, LD50 vs. 1/100^th^ of LD50 p<0.000, LD50 vs. control p<0.000).
(TIFF)

S2 Fig Lysozyme-like activity in the haemolymph of honeybees after being exposed to different *E*. *faecalis* strains and concentrations. General antibacterial activity measured as the diameter of the lytic zone on agar plates, transformed to lysozyme equivalents (μg/ml) (ANOVA: F(4,35) = 1.200, p = 0.328). Only the exposure to MMH results in a lysozyme-like activity (mean lytic activity: MMH 1 = 72.53±55.034, MMH 0.5 = 37.19±37.
(TIFF)

S1 Data (XLSX)
