# Supplementary figures and images for: Increased survival of honeybees in the laboratory after simultaneous exposure to low doses of pesticides and bacteria
Source: PLoS One. 2018 Jan 31;13(1):e0191256. doi: 10.1371/journal.pone.0191256 (PMC5791986; doi:10.1371/journal.pone.0191256)

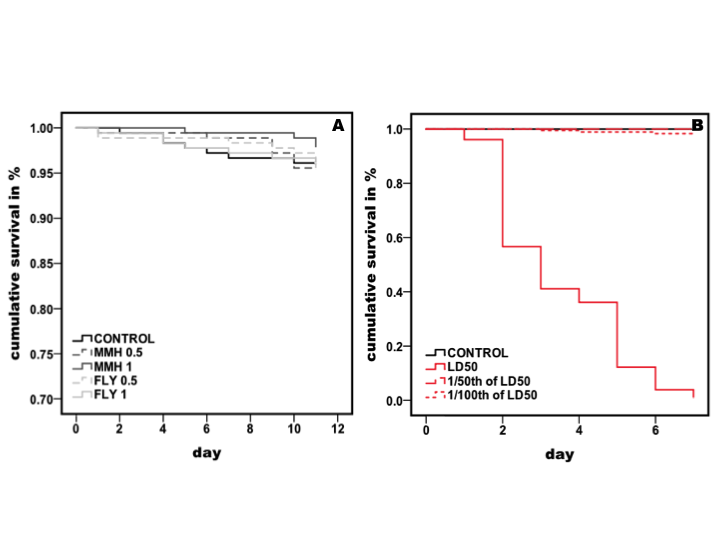

Supplement: S1 Fig — Cumulative survival of adult honeybees after (A) oral exposure with two different concentrations (OD 1 and OD 0.5) of the E. faecalis strains MMH and FLY (Kaplan-Meier log Rank: df = 4, X2 = 1.603, p = 0.808), and (B) oral exposure of the LD50 dose of the insecticide thiacloprid as well as two lower doses (1/50th of LD50 and 1/100th of LD50) (Kaplan-Meier log Rank: df = 3, X2 = 979.480, p = 0.000; multiple comparison at day 2 for LD50: LD50 vs. 1/50th of LD50 p<0.000, LD50 vs. 1/100th of LD50 p<0.000, LD50 vs. control p<0.000). (TIFF) [file pone.0191256.s001.tiff]

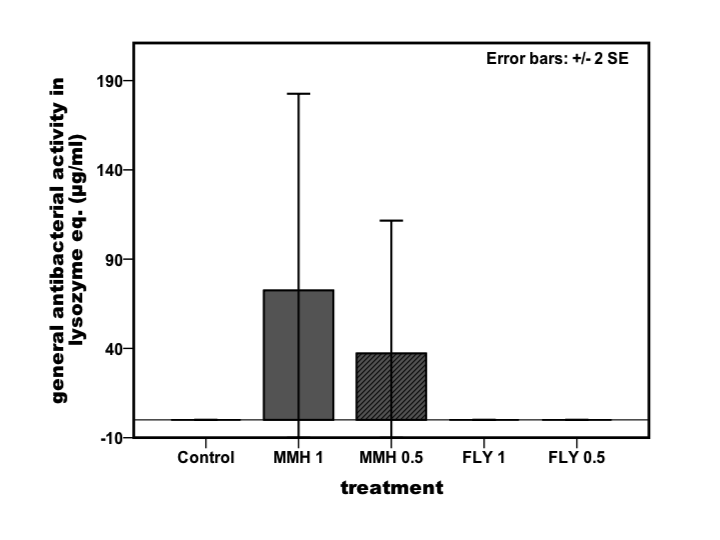

Supplement: S2 Fig — General antibacterial activity measured as the diameter of the lytic zone on agar plates, transformed to lysozyme equivalents (μg/ml) (ANOVA: F(4,35) = 1.200, p = 0.328). Only the exposure to MMH results in a lysozyme-like activity (mean lytic activity: MMH 1 = 72.53±55.034, MMH 0.5 = 37.19±37. (TIFF) [file pone.0191256.s002.tiff]
